# A G-quadruplex structure at the 5′ end of the *H19* coding region regulates *H19* transcription

**DOI:** 10.1038/srep45815

**Published:** 2017-04-03

**Authors:** Mitsuko Fukuhara, Yue Ma, Kazuo Nagasawa, Fumiko Toyoshima

**Affiliations:** 1 Department of Biosystems Science, Institute for Frontier Life and Medical Science, Kyoto University, Sakyo-ku, Kyoto 606-8507, Japan; 2 Department of Mammalian Regulatory Network, Graduate School of Biostudies, Kyoto University, Sakyo-ku, Kyoto 606-8502, Japan; 3 Department of Biotechnology and Life Science, Tokyo University of Agriculture and Technology, 2-24-16 Naka-cho, Koganei-shi, Tokyo 184-8588, Japan

## Abstract

The *H19* gene, one of the best known imprinted genes, encodes a long non-coding RNA that regulates cell proliferation and differentiation. *H19* RNA is widely expressed in embryonic tissues, but its expression is restricted in only a few tissues after birth. However, regulation of *H19* gene expression remains poorly understood outside the context of genomic imprinting. Here we identified evolutionarily conserved guanine (G)-rich repeated motifs at the 5′ end of the *H19* coding region that are consistent with theoretically deduced G-quadruplex sequences. Circular dichroism spectroscopy and electrophoretic mobility shift assays with G-quadruplex-specific ligands revealed that the G-rich motif, located immediately downstream of the transcription start site (TSS), forms a G-quadruplex structure *in vitro*. By using a series of mutant forms of *H19* harboring deletion or G-to-A substitutions, we found that the *H19*-G-quadruplex regulates *H19* gene expression. We further showed that transcription factors Sp1 and E2F1 were associated with the *H19*-G-quadruplex to either suppress or promote the *H19* transcription, respectively. Moreover, *H19* expression during differentiation of mouse embryonic stem cells appears to be regulated by a genomic *H19* G-quadruplex. These results demonstrate that the G-quadruplex structure immediately downstream of the TSS functions as a novel regulatory element for *H19* gene expression.

The *H19* gene encodes a ~2.5 kb transcript that is capped, spliced, and polyadenylated. *H19* RNA lacks an evolutionarily conserved open reading frame but has a conserved secondary RNA structure indicating it has a functional role as a non-coding RNA^1^,^2^. It has been established that the first exon of *H19* encodes microRNAs: miR-675-3p and miR-675-5p^3^. *H19* RNA is widely expressed in embryonic and extra-embryonic tissues. Shortly after birth, however, *H19* RNA expression is drastically repressed in the majority of tissues and is sustained only in specific organs, including mammary gland, uterus, cardiac and skeletal muscles^4^,^5^,^6^,^7^,^8^. Mice lacking *H19* are viable and fertile with an overgrowth phenotype at birth^9^,^10^. Recent studies have revealed the physiological functions of *H19* and miR-675, such as adult hematopoietic stem cell quiescence^11^, skeletal muscle cell differentiation^12^,^13^, and limitation of placental growth^14^.

Upon tumorigenesis, the expression of *H19* becomes dysregulated. *H19* is highly expressed in various cancers, such as breast^6^,^15^, lung^16^, bladder^17^,^18^ and colon cancers^19^,^20^. In addition, *H19* enhances tumor cell proliferation, colony formation and tumor metastasis^21^,^22^,^23^,^24^,^25^, indicating a proto-oncogenic function of *H19* and/or miR-675. Therefore, the regulation of *H19* gene expression appears to be critical for cell fate decisions and tissue homeostasis. The *H19* gene is under the control of genomic imprinting, whereby *H19* is expressed only from the maternal allele. It has been established that monoallelic expression of *H19* genes is regulated by a differentially methylated region (DMR) located between −2 kb and −4 kb upstream of the *H19* gene locus^26^,^27^,^28^. Outside of the context of genomic imprinting, however, little is known about how *H19* gene expression is regulated, and there is limited information on the genomic structure of *H19* gene.

The G-quadruplex is a non-canonical B-DNA structure, consisting of stacked square planar arrays of a guanine (G)-tetrad complexed with a monovalent cation such as Na^+^ and K^+^ (reviewed in refs 29 and 30). G-quadruplex structures are found in telomeres, promoters and regions proximal to transcription start sites (TSS) of genes^31^,^32^,^33^,^34^. Accumulating evidence has revealed a pivotal role of the DNA G-quadruplex in telomere maintenance^35^,^36^, replication^37^,^38^, chromosome fragility^39^,^40^, and transcriptional regulation of cancer related genes, including c-Myc, K-Ras, and YY1^41^,^42^,^43^.

In this report, we identify conserved G-rich motifs located immediately downstream of the *H19* TSS that forms a G-quadruplex structure. This G-quadruplex structure regulates *H19* transcription through binding to transcription factors Sp1 and E2F1. This study demonstrates for the first time the regulation of *H19* gene expression via G-quadruplex structure within its coding region.

## Methods

### Circular dichroism spectroscopy

DNA oligonucleotides (Japan Bio Services Co.) were dissolved in buffer (50 mM Tris-HCl 20 mM KCl) at concentration of 10 μM. Before analysis, the oligonucleotides were heated at 95 °C for 5 min, then slowly cooled down to room temperature, and incubate overnight. Circular dichroism (CD) spectra were recorded on a J-720 spectropolarimeter (JASCO, Tokyo, JAPAN) using a quartz cell of 1 mm optical path length and an instrument scanning speed of 500 nm/min with a response time of 1 s, and over a wavelength range of 220–320 nm. Finally CD spectra are representative of five averaged scans taken at 25 °C, then a stepwise increase of 10 °C from 25 °C to 95 °C.

### Circular dichroism melting assay

A solution of the oligonucleotides was prepared in 50 mM Tris-HCl with 20 mM KCl at concentration of 10 μM. The solution was annealed at 95 °C for 5 min, then slowly cooled down to room temperature, and incubate overnight. Melting curves were obtained by monitoring the CD intensity at 260 nm on a J-720 spectropolarimeter (JASCO, Tokyo, JAPAN) by using a quartz cell of 1 mm optical path length; the temperature was changed as follows, 25 °C to 95 °C at 1.0 °C/min.

### Electrophoretic mobility shift assays (EMSA)

DNA samples were heated at 95 °C for 5 min in 10 mM Tris-HCl, pH 8.0, with or without 100 mM KCl, and slowly cooled to room temperature. Samples were electrophoresed on a 12% native polyacrylamide gel in 0.5 × TBE buffer at 100 V (constant voltage). The gels were stained using 2D-Silver stain-II (Cosmo Bio). For EMSA using a fluorescent ligand (L1BOD-7OTD)^44^, DNA samples were incubated with 100 μM L1BOD-7OTD in 10 mM Tris-HCl, pH 8.0. The fluorescent signals were detected using a Typhoon 9410 imager (Amersham). C-myc oligonucleotide (GAG GGG CGC TTA TGG GGA GGG TGG GGA GGG TGG GGA AGG TGG GGA GGA GAC) and mutant-C-myc oligonucleotide (GAG GGG CGC TTA TGC TTA CGC TCT TGA ATC TCA TGA AGG TGG GGA GGA GAC) were used as a positive and negative control, respectively^45^.

### Cell culture and transfections

HEK293T cells, HeLa cells, U2OS cells and EpH4 cells (a gift from Dr. Ernst Reichmann, University of Zurich) were cultured in DMEM (Nissui) containing 10% fetal bovine serum (Nichirei BioScience), 4 mM L-glutamine (Nacalai), 0.15% sodium bicarbonate and antibiotics. Mouse embryonic stem cells (mESCs) (OLV2-1 cells^46^, Riken BRC, AES0153) were cultured in G-MEM containing 15% fetal bovine serum, 0.1 mM non-essential amino acids, 1 mM sodium pyruvate, 0.1 mM 2-mercaptoethanol (all GIBCO) and leukemia inhibitory factor (LIF, Wako) on a 0.1% (w/v) gelatin-coated dish. For neural differentiation of mECSs by serum-free floating culture of embryoid body-like aggregates with quick reaggregation (SFEBq) culture^47^, cells were seeded in a low-adhesion 96-well plate (Sumilon Spheroid Plates, Sumitomo) at a density of 3000 cells per well in 150 μl of SFEBq medium [G-MEM with 10% KnockOut Serum Replacement (KSR, GIBCO), 90 μM non-essential amino acids, 0.9 mM sodium pyruvate, 44 μM 2-mercaptoethanol], reaggregated and cultured for up to 7 days. Culture medium was replenished every other day. For Embryoid Body (EB) assays, mESCs were seeded and reaggregated and then cultured in EB medium (G-MEM containing 15% FBS, 1 mM sodium pyruvate, 0.1 mM non-essential amino acids and 0.1 mM 2-mercaptoethanol). After culture for 3 days, cell aggregates were transferred into a 0.1% (w/v) gelatin-coated dish and cultured for another 3 days. HEK293T, EpH4, and HeLa cells were transfected using calcium phosphate, Lipofectamine LTX (Invitrogen) in Low Ca^2+^ DMEM, or PEI max (Polyscience), respectively. mESCs were transfected by using Lipofectamine2000 (Invitrogen).

### Plasmid construction

**Figure 1:**
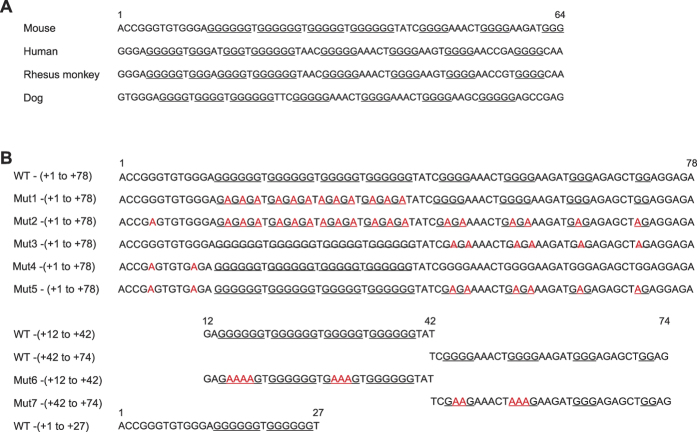
(**A**) Evolutionarily conserved G-rich sequences immediately downstream of the *H19* TSS in mammals. The guanine tandem repeats are underlined. (**B**) Oligonucleotides used in this study. Guanine-to-adenine substitutions are indicated in red.

Genomic regions of the *H19* locus (−49 to +2287 and +56 to +2287 relative to the mouse *H19* TSS) were amplified by PCR and cloned into pEF1α/pENTR (Addgene 17427)^48^. Mutagenesis within the *H19* G-quadruplex sequence was performed by PCR using the mutant oligonucleotides (see Fig. 1B). Flag-conjugated full-length mouse Sp1, NCL and E2F1 were cloned into pcDNA3.

### siRNA experiments

The siRNA sequences were as follows: Sp1-1, 5′-GGC UGG UGG UGA UGG AAU Att-3′; Sp1-2, 5′-UGG AGU GAU GCC UAA UAU Utt-3′; Luciferase, 5′-CUU ACG CUG AGU ACU UCG Att-3′. HeLa cells were transfected with siRNA by using Oligofectamine (Invitrogen).

### Dual luciferase assays

The promoter region of *H19* (−840 to +14 and −840 to +84) were amplified by PCR and cloned into pGL4.10 (Promega). HEK293T and EpH4 cells were transfected with the plasmids together with pRL [*Renilla* luciferase] plasmid (Promega). Forty-eight hours after transfection, luciferase assays were performed using the Dual-Luciferase^®^ Reporter Assay System (Promega) and an ARVO × 3 plate reader (PerkinElmer). Firefly luciferase activity was normalized against control *Renilla* luciferase activity following the manufacturer’s instructions.

### PCR, and quantitative RT-PCR (qRT-PCR)

Total DNA was isolated using phenol:chloroform and ethanol and then subjected to PCR (rTaq DNA polymerase, TOYOBO). The transfection efficiency was analyzed by PCR using a following primer pair setting on vector backbone (pEF1α/pENTR): 5′-CGG TTG CAT TCG ATT CCT GT-3′ and 5′-TTC CGA CTC GTC CAA CAT CA-3′. Total cellular RNA was extracted using an RNeasy mini kit (Qiagen). cDNAs were prepared using 0.4-1 μg of RNA and M-MLV reverse transcriptase (Invitrogen). qRT-PCR was performed with a KAPA SYBR FAST qPCR kit (NIPPON Genetics) on an ABI7500 or StepOnePlus (Applied Biosystems) and analyzed using the accompanying software. Primer pairs used for amplification were as follows: mouse *H19* Fw, 5′-CAT TCT AGG CTG GGG TCA AA-3′; mouse *H19* Rev, 5′-GCC CTT CTT TTC CAT TCT CC-3′; human *H19* Fw, 5′-ATG GTG CTA CCC AGC TCA AG-3′; human *H19* Rev, 5′-TGT TCC GAT GGT GTC TTT GA-3′; Oct4 Fw, 5′-TCT TTC CAC CAG GCC CCC GGC TC-3′; Oct4 Rev, 5′-TGC GGG CGG ACA TGG GGA GAT CC-3′; Sox1 Fw, 5′-CCT CGG ATC TCT GGT CAA GT-3′; Sox1 Rev, 5′-GCA GGT ACA TGC TGA TCA TCTC-3′; β-actin Fw, 5′-AGG CCC AGA GCA AGA GAG-3′; β-actin Rev, 5′-GGA GAG CAT AGC CCT CGT AG-3′; G3PDH Fw, 5′-ACC ACA GTC CAT GCC ATC AC-3′; G3PDH Rev, 5′-TCC ACC ACC CTG TTG CTG TA-3′.

### Pull-down assays and western blotting

The single and double stranded biotin-labeled *H19* G-quadruplex-WT-oligonucleotides (5′-biotin-ACC GGG TGT GGG AGG GGG GTG GGG GGT GGG GGT GGG GGG TAT C-3′) or the single and double stranded biotin-labeled *H19* G-quadruplex-mutant-oligonucleotides (5′-biotin-ACC GAG TGT GGG AGA GAG ATG AGA GAT AGA GAT GAG AGA TAT C-3′) (20 μM) were heated at 95 °C for 5 min in 10 mM Tris-HCl, pH 8.0 with 100 mM KCl, and cooled to 4 °C. HeLa cells and mESCs were washed with PBS, then resuspended in binding buffer (50 mM Hepes, pH 7.3, 2 mM EGTA, 2 mM MgCl_2_, 1 mM EDTA, 15 mM NaF, 10 mM β-glycerophosphate, 10% glycerol, 100 mM KCl, 1 mM DTT, 10 μg/ml Aprotinin, 1 mM PMSF, and 1% NP-40). After incubation for 15 min on ice, the cell suspensions were sonicated (10 sec at 1.2 output, 4 cycles), and the insoluble fractions were removed by centrifugation. The obtained cell extracts were incubated with 10 μl of the oligonucleotides for 1 hour at 4 °C, followed by the addition of 20 μl streptavidin sepharose beads (GE Healthcare) pre-blocked with 1% BSA and 200 μg/ml salmon sperm DNA, and incubated for another 30 min at 4 °C. The beads were washed three times with binding buffer without NP-40 and once with PBS, and resuspended in 100 μl SDS sample buffer and boiled. Aliquots of 20 μl were subjected to western blotting using the following primary antibodies: rabbit anti-Sp1 (21962-1-AP, Protein tech), rabbit anti-NCL (H-250, Santa Cruz), rabbit anti-PARP1 (sc-7150, Santa Cruz), rabbit anti-E2F1 (sc-193, Santa Cruz), and mouse anti-α-tubulin (T6557, Sigma). Primary antibodies were detected with HRP-conjugated secondary antibodies (GE Healthcare) using ECL-Plus reagents (PerkinElmer).

### Chromatin Immunoprecipitation (ChIP)

HeLa cells were cross-linked with 1% formaldehyde at room temperature for 10 min. Glycine was added at a final concentration of 0.125 M, followed by incubation for 10 min at room temperature. The cells were washed with PBS, then lysed with SDS lysis buffer (50 mM Tris, 10 mM EDTA, 1% SDS, 1 mM PMSF, 10 μg/mL aprotinin, and 1 mM DTT) and diluted with ChIP dilution buffer (50 mM Tris, 167 mM NaCl, 1.1% Triton X-100, 0.11% sodium deoxycholate, 1 mM PMSF, 10 μg/mL aprotinin, and 1 mM DTT). Samples were sonicated, centrifuged at 15,000 rpm at 4 °C for 10 min and recover supernatant. Rabbit anti-Sp1, rabbit anti-NCL, rabbit anti-E2F1 or rabbit IgG (invitrogen) and Dynabeads M-280 Sheep anti-Rabbit IgG (life technologies) were preincubated with 1% BSA and 200 μg/μl salmon sperm DNA at 4 °C for 3 h and then added to the samples, followed by incubation at 4 °C overnight. The immunoprecipitates were washed with wash buffer A (50 mM Tris, 150 mM NaCl, 1 mM EDTA, 1% Triton X-100, 0.1% SDS, and 0.1% sodium deoxycholate) twice, wash buffer B (50 mM Tris, 500 mM NaCl, 1 mM EDTA, 1% Triton X-100, 0.1% SDS, and 0.1% sodium deoxycholate), and TE buffer (10 mM Tris, 1 mM EDTA) twice. The bound DNA was eluted with ChIP elution buffer (10 mM Tris, 300 mM NaCl, 5 mM EDTA, and 0.5% SDS) at 65 °C overnight. Eluted DNA was purified with phenol/chloroform and ethanol, and then subjected to qPCR analysis. qPCR was performed using the following primers: 5′-GCA CCT TGG ACA TCT GGA GT-3′ and 5′-TTC TTT CCA GCC CTA GCT CA-3′.

### Construction of *H19* G-quadruplex disrupted mESC lines

Genomic mutagenesis of the *H19* G-quadruplex sequence was performed using the Crispr/Cas9 system. Briefly, mESCs were co-transfected with a plasmid encoding Cas9 and a guide RNA (Addgene 44248)^49^ targeting the first exon of *H19* (5′-GAG GAG AGT CGT GGG GTC CG-3′) and pEF1α/pENTR containing Mut2-*H19* (−840 to +2287) (see Fig. 1B) using Lipofectamine 2000 (Invitrogen). The transfected cells were selected by incubation with puromycin and clonally expanded. Bi-allelic mutations were confirmed by genomic sequencing. Two lines of mESCs harboring genomic Mut2 mutation were established and used for the analysis (Mut2-G4-a cell and Mut2-G4-b cell).

### Statistical analysis

All data are representative of at least three independent experiments. *P* values were calculated by applying Dunnett’s multiple-comparison test or two-tailed t-test. Data are presented as the mean ± standard error of the mean.

## Results

### The G-rich motifs located immediately downstream of the *H19* TSS form a G-quadruplex structure

**Figure 2:**
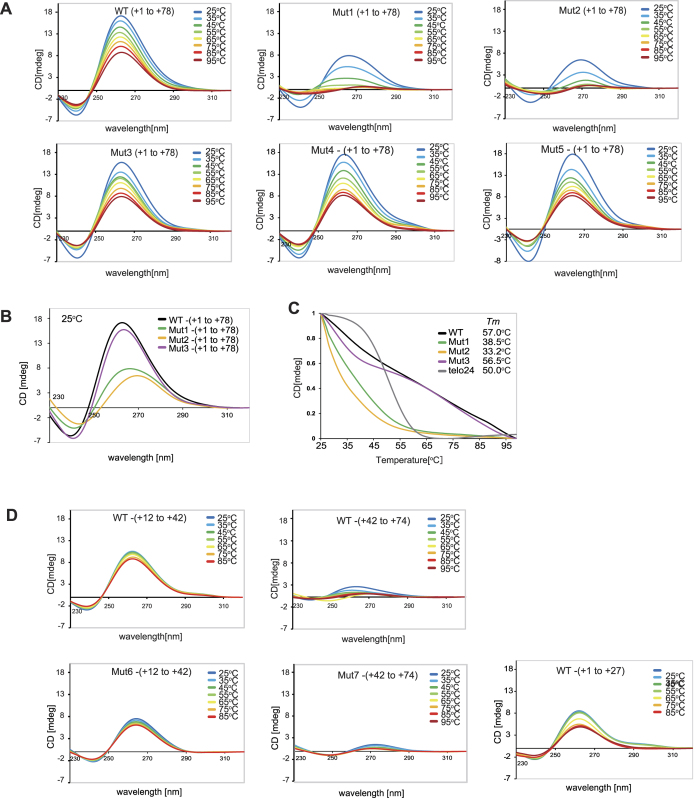
Evidence for G-quadruplex formation of the *H19* G-rich sequences by CD spectroscopy. (**A**) CD spectra of indicated oligonucleotides (regions between +1 to +78 of the mouse *H19* gene). CD spectra were measured in the presence of 20 mM KCl at various temperatures between 25 °C to 95 °C. (**B**) CD spectra of indicated oligonucleotides at 25 °C. (**C**) *T*_m_ values for each oligonucleotide were calculated from CD melting assay. Telo24 (TTAGGG)_4_, a well known G-quadruplex sequence^66^, was used as a control. (**D**) CD spectra of indicated oligonucleotides (regions between +12 to +42, +42 to +74 or +1 to +27 of the mouse *H19* gene) were measured in the presence of 20 mM KCl at various temperatures between 25 °C to 95 °C.

*H19* harbors G-rich sequences immediately downstream of the TSS, which are conserved in mammalian species (Fig. 1A). The G-rich sequence in the region between +14 and +39 of mouse *H19* displays a G-score of 84, calculated by QGRS Mapper (Max length: 30, Min G-group: 2, Loop size: 0 to 36), a software that provides information on the composition of putative G-quadruplex forming G-rich sequences^50^, suggesting that this region forms a G-quadruplex structure. To assess this possibility, we prepared single-stranded oligonucleotides corresponding to the regions between +1 to +78, +12 to +42, +42 to +74 and +1 to +27 of mouse *H19*, and analyzed them by circular dichroism (CD) spectroscopy. The CD spectrum of the oligonucleotide for the region between +1 to +78 was characteristic for parallel G-quadruplex structures^51^ in the presence of 20 mM KCl [Fig. 2A, WT-(+1 to +78)]. A similar CD spectrum pattern was observed when the guanine (G) nucleosides within the region between +42 to +78, +1 to +12 or both were substituted by adenine (A) [Figs 1B and 2A, Mut3-, Mut4-, and Mut5-(+1 to +78)]. However, G-to-A substitutions throughout the region between +1 to +78 [Fig. 1B, Mut2-(+1 to +78)] or within the region between +12 to +42 [Fig. 1B, Mut1-(+1 to +78)], resulted in disruption of the CD spectrum patterns [Fig. 2A, Mut1-(+1 to +78) and Mut2-(+1 to +78)]. The CD spectra at 25 °C (Fig. 2B) and at their respective *T*_m_ values (Fig. 2C) support the notion that WT-(+1 to +78) and Mut3-(+1 to +78), but not Mut1-(+1 to +78) or Mut2-(+1 to +78), form G-quadruplex structures. In addition, the oligonucleotide for the region between +12 to +42 [Figs 1B and 2D, WT-(+12 to +42)] exhibited a characteristic spectrum for parallel G-quadruplex structures. In contrast, the oligonucleotide for the region between +42 to +74, as well as the mutant oligonucleotides with G-to-A substitutions in this region, barely formed G-quadruplex structures [Figs 1B and 2D, WT-(+42 to +74) and Mut7-(42 to +72)]. The oligonucleotides for the region between +1 to +27 partially exhibited a characteristic spectrum for parallel G-quadruplex structures [Figs 1B and 2D, WT-(+1 to +27)]. These results suggest that the region between +12 to +42 within the *H19* gene forms a DNA G-quadruplex structure. The region between +1 to +27 is capable of forming a G-quadruplex structure in the short oligonucleotides, but the region between +1 to +12 is dispensable for G-quadruplex structure formation in the longer oligonucleotides.

**Figure 3:**
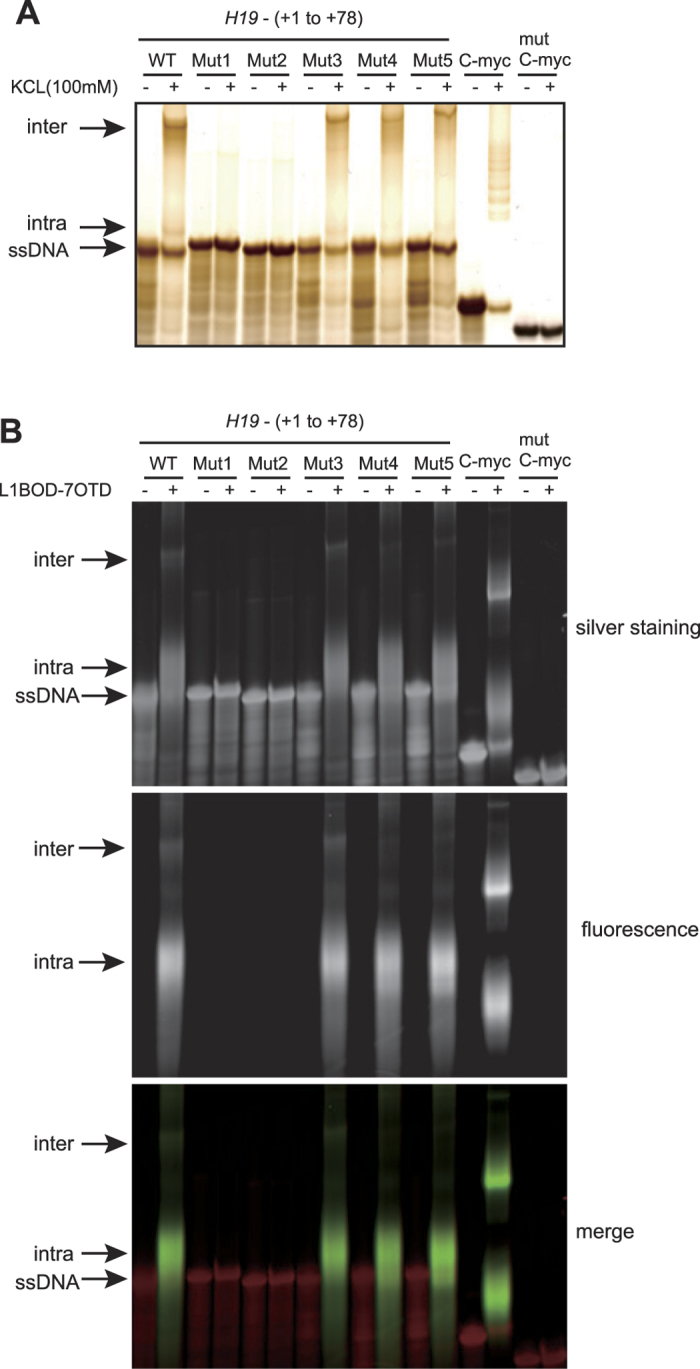
Evidence for G-quadruplex formation of *H19* G-rich sequences by EMSA incorporating a G-quadruplex stabilizing compound. (**A**) Oligonucleotides were incubated in the presence or absence of 100 mM KCl. After electrophoresis, the gel was subjected to silver staining. (**B**) Oligonucleotides were incubated with or without L1BOD-7OTD, a fluorescein-conjugated G-quadruplex stabilizing compound and electrophoresed. Silver stained images and fluorescent signals are shown. C-myc and mutant-C-myc oligonucleotides (51 nt) were used as a positive and negative control, respectively.

We next performed an EMSA to investigate the conformational change of the *H19* G-rich sequence resulting from the addition of physiologically relevant levels of KCl (100 mM). In the absence of 100 mM KCl, WT-, Mut1-, Mut2-, Mut3-, Mut4- and Mut5-(+1 to +78) oligonucleotides exhibited a single band at the corresponding single-stranded (ss) DNA size (Fig. 3A, KCl−). In the presence of 100 mM KCl, however, WT-, Mut3-, Mut4- and Mut5-(+1 to +78) oligonucleotides, but not Mut1- and Mut2-(+1 to +78) oligonucleotides, exhibited additional slow-migrating bands corresponding to inter- and intra-molecular G-quadruplex structures (Fig. 3A, KCl+). We then used the fluorescein-conjugated compound L1BOD-7OTD, a derivative of telomestatin, which specifically interacts with and stabilizes G-quadruplex structures^44^. In the presence of L1BOD-7OTD, WT-, Mut3-, Mut4-, and Mut5-(+1 to +78) oligonucleotides, but not Mut1- and Mut2-(+1 to +78) oligonucleotides, again exhibited the additional slow-migrating bands even in the absence of KCl (Fig. 3B, silver staining). We also detected fluorescence signals in the slow-migrating bands (Fig. 3B, fluorescence). Consistent with the results of CD assay, the short form of oligonucleotides WT-(+12 to +42) exhibited slow-migrating bands with fluorescence signals (Supplementary Figure 1), confirming the ability of this sequences to form G-quadruplex structures. WT-(+1 to +27) also partially exhibited slow-migrating bands with fluorescence signals (Supplementary Figure 1), although this region is dispensable for G-quadruplex structure formation in longer stretch of DNA sequences. Notably, Mut6-(+12 to +42), the short form of mutant oligonucleotides with G-to-A substitutions in the central G-quadruplex sequence, exhibited the partially disrupted CD spectra but still forms G-quadruplex [Figs 1B and 2D, Mut6-(+12 to +42)]. However, the EMSA analysis with L1BOD-7OTD showed that Mut6-(+12 to +42) formed an intermolecular, rather intramolecular, G-quadruplex structure (Supplementary Figure 1). These results, taken together, demonstrate that the G-rich sequence within the region between +12 to +42 of *H19* forms a G-quadruplex structure *in vitro*.

### The *H19* G-quadruplex regulates *H19* gene transcription

**Figure 4:**
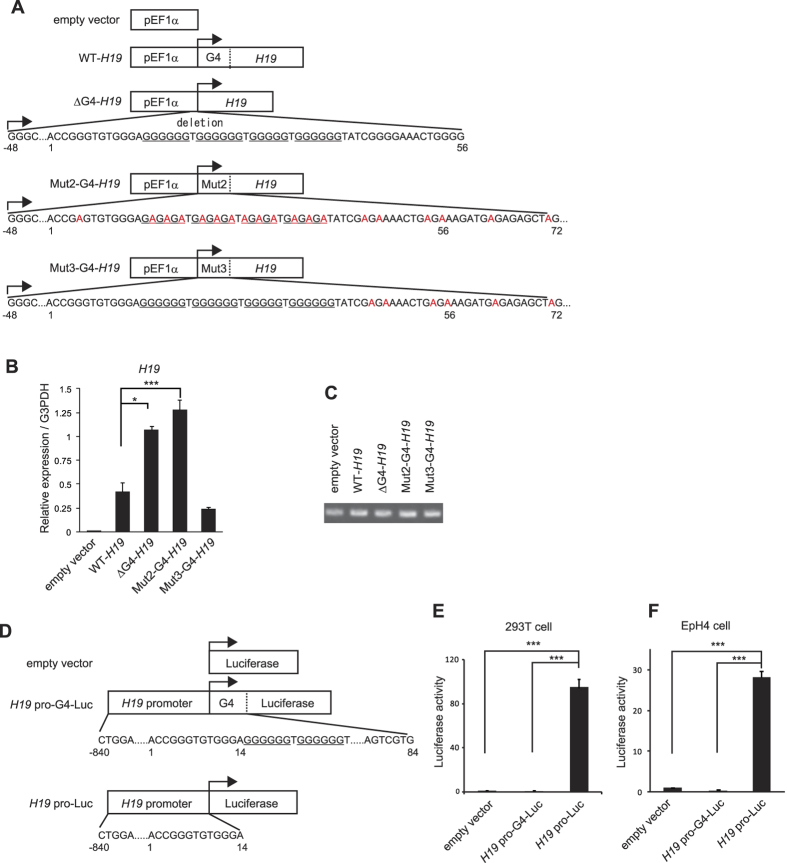
*H19* G-quadruplex suppresses transcription. (**A**) Schematic diagram of the *H19* expression vectors used in this study. The full length *H19* gene (WT-*H19*), G-quadruplex sequence-truncated *H19* gene (∆G4-*H19*), or *H19* gene with Mut2 or Mut3 G-quadruplex sequence (Mut2-G4-*H19*, Mut3-G4-*H19*) were driven by the EF1α promoter. (**B**) qPCR analysis of *H19* RNA levels in 293T cells transfected with the indicated plasmids. Values are normalized against G3PDH. (**C**) PCR analysis for transfection efficiency of the plasmids in (**B**) using a primer pair specific to the vector backbone. (**D**) Schematic diagram of the luciferase reporter plasmids. *H19* G-quadruplex sequence was inserted just upstream of the *luciferase* gene (Luc). *Luc* or *G-quadruplex-Luc* (G4-Luc) was driven by the *H19* promoter (the region between −840 to +14). (**E**,**F**) 293 T cells (**E**) or EpH4 cells (**F**) were transfected with the luciferase reporter plasmids together with the control RL-SV40 plasmid, and the luciferase activities measured. Values are normalized against the activity of co-transfected *Renilla* luciferase. (**B**,**E**,**F**) mean ± s.d. from three experiments; *P < 0.05, ***P < 0.005 analyzed by Dunnett’s multiple-comparison test.

To determine whether the G-quadruplex structure at the *H19* TSS regulates *H19* gene transcription, we constructed a series of plasmids encoding mouse WT-*H19*, Mut2-G4-*H19*, Mut3-G4-*H19*, or ∆G4-*H19*, where the +1 to +56 region of *H19* was deleted, under the control of the EF1α promoter (Fig. 4A). 293T cells were transfected with each plasmid and the expression levels of *H19* RNA were analyzed by qRT-PCR. PCR analysis confirmed the same degree of transfection efficiency of the plasmids (Fig. 4C). The results showed that the expression levels of *H19* RNA were significantly higher in the cells transfected with ∆G4-*H19* and Mut2-G4-*H19* compared with the WT-*H19*-transfected cells (Fig. 4B) at the various concentrations of the plasmids (Supplementary Figure 2). Mut3-G4-*H19* exhibited the similar level of *H19* RNA as WT-*H19* (Fig. 4B), suggesting that the G-rich sequences within the region between +43 to +78 is dispensable for regulating *H19* gene transcription. Next, we constructed luciferase assay vectors, in which the *H19* promoter element together with G-quadruplex sequence (−840 to +84) (*H19* pro-G4-Luc) or the *H19* promoter element alone (−840 to +14) (*H19* pro-Luc) was fused to the luciferase-coding sequence (Fig. 4D). We transfected these vectors into 293T or EpH4 cells, and found that luciferase activity was much higher in the cells transfected with *H19* pro-Luc compared with that of *H19* pro-G4-Luc or the control plasmid-transfected cells (Fig. 4E and F). These results indicate that the *H19* G-quadruplex sequence in the regions between +1 to +42 has a function to suppress *H19* gene transcription.

### Identification of proteins associated with the *H19* G-quadruplex

**Figure 5:**
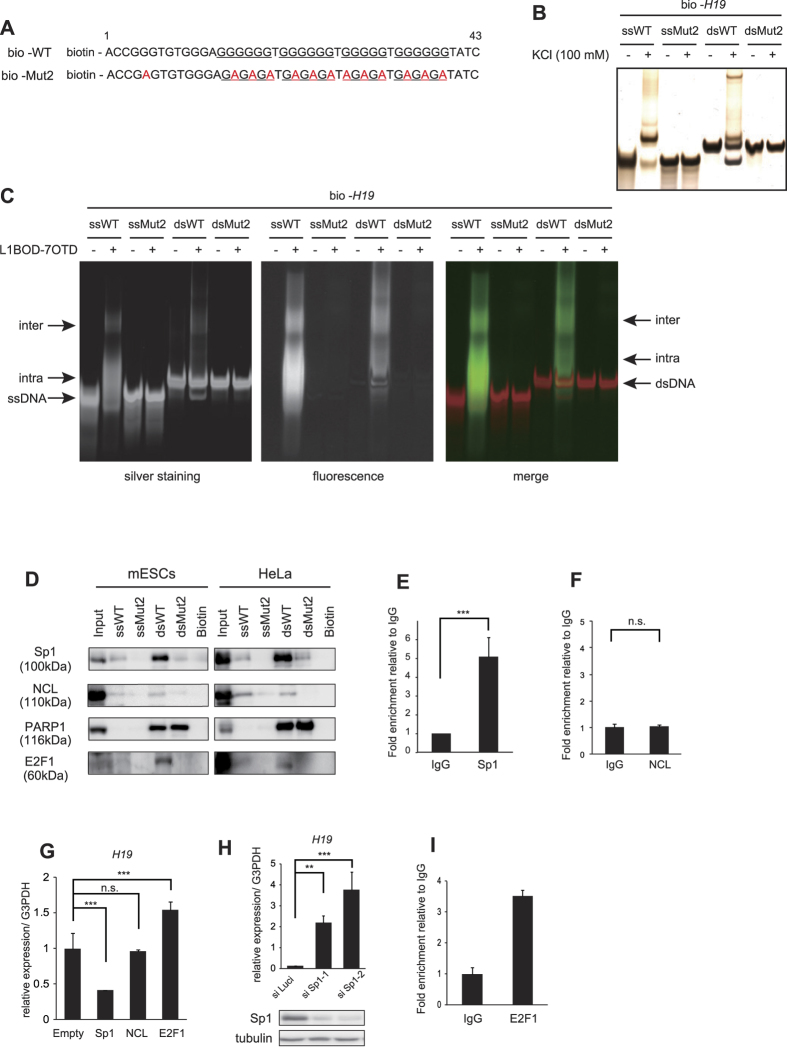
Identification of *H19* G-quadruplex associated factors. (**A**) Biotin-labeled oligonucleotide sequences used in the pull-down assays. (**B**) Single-stranded (ss) or double-stranded (ds) biotin-labeled oligonucleotides in (**A**) were prepared by heating at 95 °C for 5 min, followed by cooling to 4 °C. The oligonucleotides were incubated with or without 100 mM KCl, electrophoresed, and subjected to silver staining. (**C**) Each oligonucleotide prepared as in (**B**) was incubated with or without L1BOD-7OTD, and electrophoresed. Silver stained images and fluorescent signals are shown. (**D**) Western blot analysis of Sp1, NCL, PARP1 and E2F1 for the pull-down assay of each oligonucleotide. The input is 10% of the total fraction used for each sample. Uncropped images are in Supplementary Figure 6A. (**E**,**F**) ChIP-qPCR analysis against Sp1 (**E**), NCL (**F**) or control IgG in HeLa cells by using primer pairs for amplifying the region between +113 and +282. The obtained qPCR values are normalized by input DNA. Fold enrichment relative to IgG is shown. (**G**) qPCR analysis for endogenous *H19* RNA levels in HeLa cells transiently expressing Sp1, NCL and E2F1. Values are normalized against G3PDH. (**H**) qPCR analysis for endogenous *H19* RNA levels in HeLa cells depleted with Sp1 by siRNA. Western blot analysis of Sp1 and control tubulin are shown in the bottom. Uncropped images are in Supplementary Figure 6B. (**I**) ChIP-qPCR analysis against E2F1 or control IgG in HeLa cells ectopically expressing E2F1. The obtained qPCR values are normalized by input DNA. Fold enrichment relative to IgG is shown. (**E**–**I**) mean ± s.d. from three (**E**–**H**) or two (**I**) independent experiments; **P < 0.01, ***P < 0.005 analyzed by two-tailed t-test (**E**,**F**) or Dunnett’s multiple-comparison test (**G**,**H**).

To gain insight into the molecular mechanisms, we next determined the *H19* G-quadruplex-associate proteins. To this end, we performed a pull-down assay using the biotinylated WT- and Mut2-oligonucleotides. As the region between +43 to +78 was dispensable for suppressing *H19* gene transcription (see Fig. 4B), we used the biotinylated WT- and Mut2-oligonucleotides for the region between +1 to +43 of *H19* for a pull-down assay (Fig. 5A, bio-WT and bio-Mut2, respectively). We confirmed that both single- and double-stranded bio-WT, but not bio-Mut2, exhibit mobility-shift bands in EMSA in the presence of 100 mM KCl (Fig. 5B) or L1BOD-7OTD (Fig. 5C). We incubated the single- or double-stranded biotinylated oligonucleotides with the cell lysates in the presence of 100 mM KCl. By using whole cell lysates from HeLa cells and mouse embryonic stem cells (mESCs), we examined the association of the oligonucleotides with proteins that have been reported to interact with G-quadruplex, including Sp1^52^,^53^,^54^, Nucleolin (NCL)^52^,^53^,^55^, and Poly(ADP-ribose) polymerase-1 (PARP1)^56^,^57^,^58^. We found that Sp1 and NCL bound to bio-WT, but not bio-Mut2 (Fig. 5D). On the other hand, PARP1 bound to both bio-WT and bio-Mut2 double-stranded oligonucleotides, but not single-stranded oligonucleotides (Fig. 5D), indicating that PARP1 is associated with double-stranded oligonucleotides in a DNA sequence-independent manner. ChIP-qPCR analysis showed binding of endogenous Sp1, but not NCL, to the genomic region of *H19* G-quadruplex (Fig. 5E and F). These observations indicate that Sp1 is associated with the *H19* G-quadruplex both *in vitro* and *in vivo*. Notably, Sp1 bound to bio-WT more efficiently in the lysates prepared from the G1/S phase-synchronized cells than that from M phase-synchronized cells (Supplementary Figure 3B), suggesting the cell cycle-dependent association of Sp1 with the *H19* G-quadruplex. We found that the ectopic expression of Sp1 in HeLa cells resulted in the decrease of endogenous *H19* RNA level (Fig. 5G). Conversely, knockdown of Sp1 by siRNA resulted in upregulation of the *H19* RNA level (Fig. 5H), indicating that Sp1 suppresses *H19* gene transcription. We further found that E2F1, which is reported to regulate *H19* gene transcription^23^, also bound to single- and double-stranded bio-WT, but not bio-Mut2 in a pull-down assay (Fig. 5D). Importantly, the ectopically expressing E2F1 in HeLa cells binds to the genomic region of *H19* G-quadruplex (Fig. 5I) and increased the endogenous *H19* RNA level (Fig. 5G). The ectopic expression of NCL had no effect on the *H19* RNA level (Fig. 5G). These results taken together indicate that, through binding to the *H19* G-quadruplex, Sp1 and E2F1 regulate *H19* transcription in an opposite way; Sp1 suppresses whereas E2F1 promotes *H19* gene transcription.

### Genomic *H19* G-quadruplex regulates *H19* transcription during mESC differentiation

**Figure 6:**
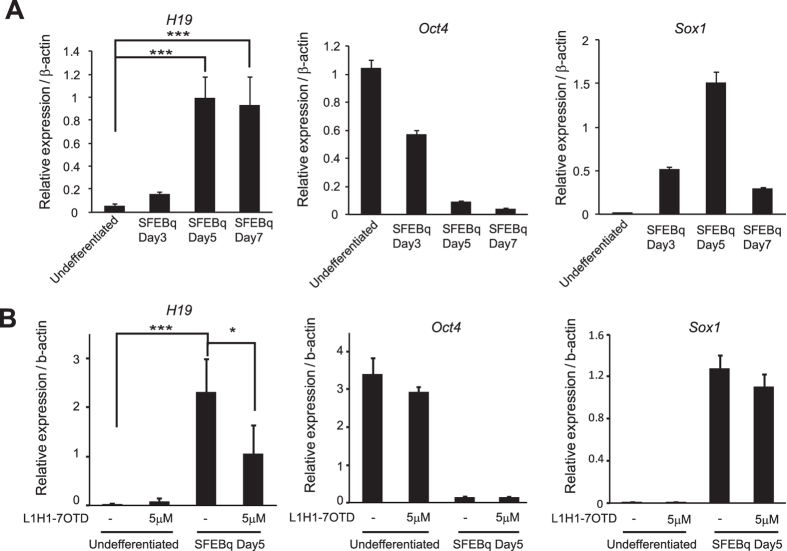
L1H1-7OTD suppresses *H19* transcription during mESC differentiation. (**A**) qRT-PCR analysis of RNA levels of *H19, Oct4* and *Sox1* during neural differentiation of mESCs by SFEBq at day 0, day 3, day 5 and day 7. (**B**) mESCs were differentiated in SFEBq medium in the presence or absence of L1H1-7OTD (5 μM). The qPCR analysis of *H19, Oct4* and *Sox1* RNA levels at differentiation Day 0 or Day 5 are shown. Values are normalized against β-actin. mean ± s.d. from three experiments; *P < 0.05, ***P < 0.005 analyzed by Dunnett’s multiple-comparison test.

**Figure 7:**
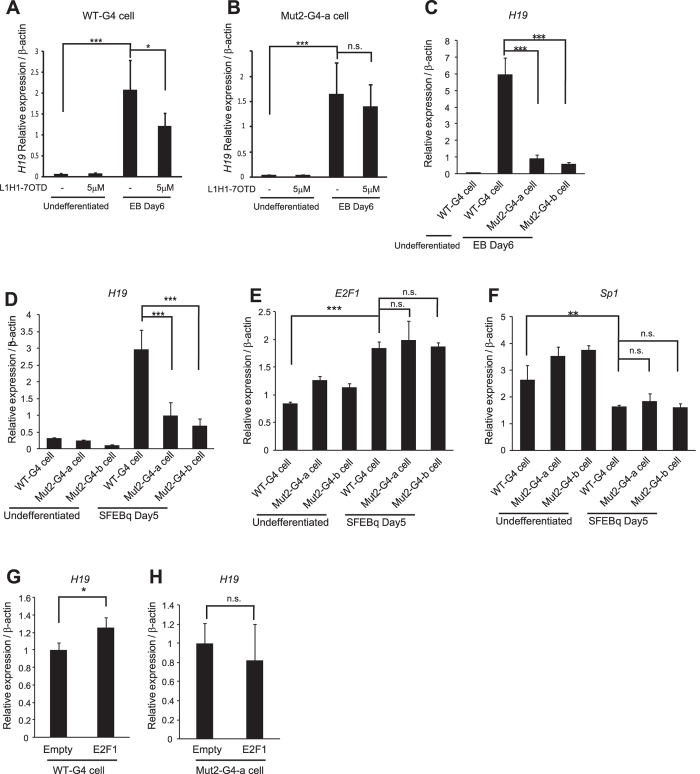
The genomic *H19* G-quadruplex sequence regulates *H19* expression. (**A**,**B**) Control mESCs (WT-G4 cell) or Mut2-G4-mESCs (Mut-G4-a cell)were cultured in mESC maintenance medium (undifferentiated) or differentiated in EB medium for 6 days (EB Day6) in the presence or absence of L1H1-7OTD (5 μM). The qPCR analyses of *H19* RNA levels are shown. (**C**) qRT-PCR analysis of *H19* RNA levels in WT- and Mut-G4-a, or -b cells in EB culture on day 6. (**D**–**F**) qRT-PCR analysis for the RNA levels of *H19* (**D**), *E2F1* (**E**) and *Sp1* (**F**) in WT- and Mut2-G4 cells in mECS maintenance medium (undifferentiated) or in SFEBq culture on day 5. (**G**,**H**) qRT-PCR analysis for *H19* RNA levels in WT- (**G**) and Mut2-G4 cells (**H**) transfected with or without the E2F1-coding plasmids. (**A**–**H**) Values are normalized against β-actin. mean ± s.d. from three experiments; *P < 0.05, **P < 0.01, ***P < 0.005 analyzed by Dunnett’s multiple-comparison test (**A**–**F**) and two-tailed t-test (**G**,**H**).

It has been reported that the expression level of *H19* RNA increases during differentiation of mESCs^8^,^59^,^60^,^61^. Consistently, we observed that levels of *H19* RNA increased during neural differentiation of mESCs by the SFEBq method on Day 5 and Day 7, while expression of a pluripotent gene, *Oct4*, and a neural-progenitor-specific gene, *Sox1*, are decreased and transiently increased, respectively (Fig. 6A). We found that addition of the compound, L1H1-7OTD, which can bind and stabilize G-quadruplex structures^62^, into the SFEBq differentiation media significantly decreased *H19* RNA levels, without affecting the expression levels of *Oct4* or *Sox1* on Day 5 (Fig. 6B). L1H1-7OTD also decreased *H19* RNA levels in HeLa cells and U2OS cells (Supplementary Figure 4). This indicates *H19* G-rich sequence forms a functional G-quadruplex structure in genome. Similarly, when mESCs were differentiated into the three germ layers by the EB culture method, *H19* RNA levels were increased at differentiation Day 6, and this increase was significantly attenuated when L1H1-7OTD was added to the differentiation medium (Fig. 7A). To investigate a functional relevance of the genomic *H19* G-quadruplex structure, we established the mESC lines where the genomic *H19* G-quadruplex sequence was replaced by the *H19*-Mut2-G4 sequence (Mut2-G4 cell) (see Fig. 1B). The Mut2-G4 cells proliferated efficiently comparable to WT-G4 cells in the mESC maintenance medium (data not shown) and were capable of differentiating into all three-germ layers, including nestin-expressing ectodermal cells, α-fetoprotein (α-FP)-expressing endodermal cells, and α-smooth muscle actin (α-SMA)-expressing mesodermal cells (Supplementary Figure 5A), indicating Mut2-G4 cells retain self-renewal ability and pluripotency. Mut2-G4 cells also properly underwent neural differentiation by the SFEBq method (Supplementary Figure 5B). We found that in the Mut2-G4 cells, the L1H1-7OTD-induced downregulation of *H19* RNA level was attenuated during differentiation (Fig. 7B), indicating that L1H1-7OTD suppresses *H19* gene transcription through binding to the genomic *H19* G-quadruplex structure. Inconsistently, however, *H19* RNA levels in Mut2-G4 cells were significantly lower than that in WT-G4 cells both in EB culture on Day6 (Fig. 7C) and in SFEBq culture on Day5 (Fig. 7D), suggesting an *H19* transcription-promoting function of the *H19* G-quadruplex sequence. Consistent with this notion, the level of E2F1, which promotes *H19* gene transcription (see Fig. 5G), became increased, whereas Sp1, which suppresses *H19* gene transcription (see Fig. 5G and H), was decreased during neural differentiation of both WT- and Mut2-G4 cells. Furthermore, ectopic expression of E2F1 significantly increased the endogenous *H19* RNA level in WT-G4 cells, but not in Mut2-G4 cells in the mESC maintenance medium. These results demonstrate that the genomic *H19* G-quadruplex structure immediately downstream of TSS regulates *H19* transcription during mESC differentiation in a dual opposite way.

## Discussion

The *H19* gene is located 200 kb downstream of the *Insulin-like growth factor 2 (Igf2*) gene on chromosome 7 in mice and 11p15.5 in humans^63^. The *H19*-*Igf2* locus is under the control of genomic imprinting, whereby *H19* is expressed from the maternal allele and *Igf2* is expressed from the paternal allele. The mechanism of the monoallelic expression of *H19* by the epigenetic modification within DMR is well established and the methylation pattern within DMR is generally maintained indefinitely. However, the mechanism that explains the differential expression of *H19* among cell types or tissues, which would be relevant to cell differentiation condition, remains unclear.

In this report, we show that *H19* gene transcription is regulated by a G-quadruplex which is located at the region immediately downstream of *H19* TSS. *H19* expression is increased during mESC differentiation, which is attenuated by the G-quadruplex stabilizing compound L1H1-7OTD in WT-G4 mESCs but not in Mut2-G4 mESCs, indicating the functional *H19* G-quadruplex-mediated *H19* transcription regulation. It has been reported that the monoallelic expression of *H19* is maintained during ESC differentiation^64^. Therefore, the *H19* G-quadruplex-mediated *H19* transcription regulation during mESC differentiation seems independent of genomic imprinting. In Mut2-G4 cells, *H19* expression level is lower than that in WT-G4 cells, suggesting the promoting role of G-quadruplex in *H19* transcription. Consistently, our results show that E2F1 binds to the *H19* G-quadruplex, and promotes *H19* transcription. Notably, however, the *H19* expression level was partially upregulated in the Mut2-G4 cells during differentiation (see Fig. 7B and D). Therefore, in addition to the G-quadruplex-mediated mechanism, *H19* gene transcription would also require *H19* promoter activation during mESC differentiation. On the other hand, our results show that Sp1 also binds to the *H19* G-quadruplex, and suppresses *H19* transcription. It is worth to note that the expression levels of E2F1 and Sp1 are increased and decreased, respectively, during mESC differentiation (see Fig. 7E and F). Therefore, the balance of E2F1/Sp1 expression levels would determine the function of the *H19* G-quadruplex on *H19* gene transcription regulation.

How E2F1/Sp1 regulates *H19* gene transcription through the *H19* G-quadruplex remains open question. Sp1 is known to recruit a large number of proteins including transcription initiation complex and transcription repressor complex^65^. Our data show that Sp1 acts as *H19* transcription repressor in conjunction with *H19* G-quadruplex. Although we could not determine whether Sp1 recognizes the G-quadruplex structure or Sp1-target sequence within the *H19* G-rich motif, it would be possible that Sp1 recruits transcription repressor complexes to the *H19* G-quadruplex to suppress *H19* transcription. It has previously shown that E2F1 binds to *H19* promoter region^23^. We show that E2F1 is associated with the *H19* G-quadruplex downstream of *H19* TSS. It would be interesting to determine which region or both plays a pivotal role in promoting *H19* transcription.

This study describes a regulatory mechanism for *H19* gene transcription via the G-quadruplex that has not been described before. Putative G-quadruplex sequences are distributed throughout the genomic regions of non-coding RNA; therefore, these G-quadruplexe structures would function as regulatory elements of transcription. *H19* RNA is highly expressed in various cancers and plays a proto-oncogenic function in several tumors. Therefore, our findings implicate that G-quadruplex-mediated transcription regulation of *H19* gene would be an effective target for anti-cancer agent.

## Additional Information

**How to cite this article**: Fukuhara, M. *et al*. A G-quadruplex structure at the 5′ end of the *H19* coding region regulates *H19* transcription. *Sci. Rep.* **7**, 45815; doi: 10.1038/srep45815 (2017).

**Publisher's note:** Springer Nature remains neutral with regard to jurisdictional claims in published maps and institutional affiliations.

## Supplementary Material

Supplementary Information
