# Supplementary material for: A G-quadruplex structure at the 5′ end of the H19 coding region regulates H19 transcription
Source: Sci Rep. 2017 Apr 3;7:45815. doi: 10.1038/srep45815 (PMC5377947; doi:10.1038/srep45815)
Supplement: Supplementary Information [file srep45815-s1.pdf]

Supplementary Materials for

A G-quadruplex structure at the 5′ end of the *H19* coding region regulates *H19* transcription

Mitsuko Fukuhara[1,2], Yue Ma[3], Kazuo Nagasawa[3], Fumiko Toyoshima[1,2]

[1] *Department of Biosystems Science, Institute for Frontier Life and Medical Science, Kyoto University, Sakyo-ku, Kyoto 606-8507, Japan*

[2] *Department of Mammalian Regulatory Network, Graduate School of Biostudies, Kyoto University, Sakyo-ku, Kyoto 606-8502, Japan*

[3] *Department of Biotechnology and Life Science, Tokyo University of Agriculture and Technology, 2-24-16 Naka-cho, Koganei-shi, Tokyo 184-8588, Japan*

Address correspondence to: Fumiko Toyoshima

e-mail: ftoyoshi@virus.kyoto-u.ac.jp

This PDF file includes :

Supplementaty Figure 1 to 6

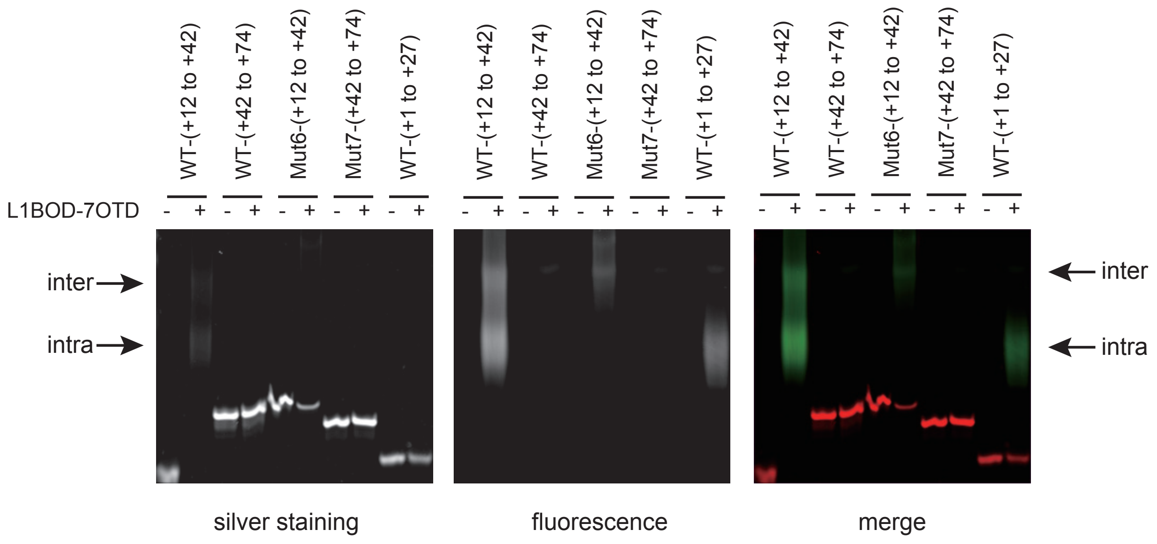

Supplementary Figure 1. EMSA analyses of the short forms of oligonucleotides for the regions downstream of *H19* TSS. Oligonucleotides were incubated with or without L1BOD-7OTD and electrophoresed. Silver stained images and fluorescent signals are shown.

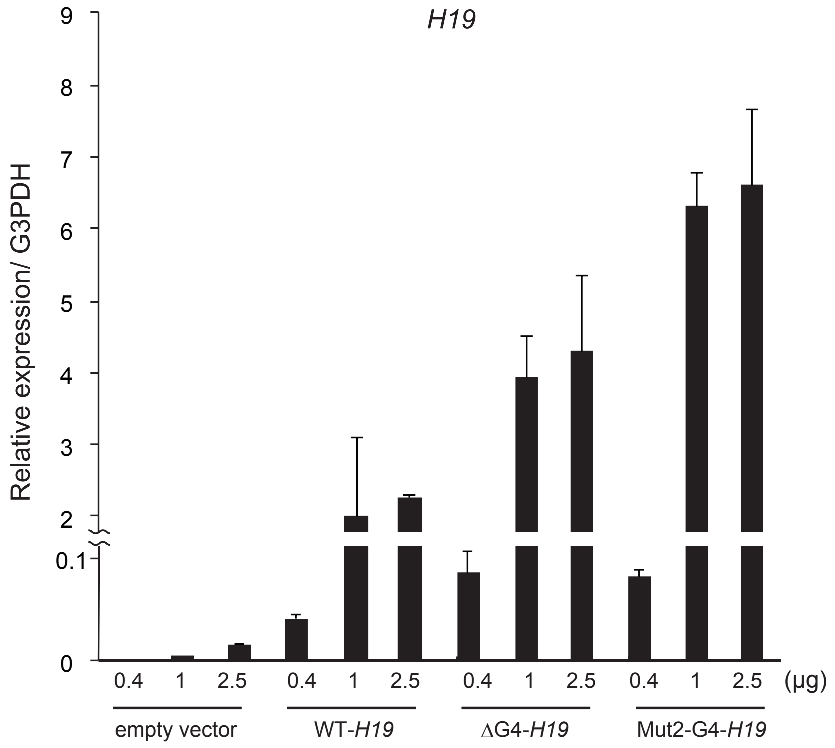

Supplementary Figure 2. qPCR analyses of *H19* RNA levels in 293T cells transfected with the indicated concentrations (0.4μg, 1μg and 2.5μg) of empty vector or the plasmids coding WT-*H19*, ΔG4-*H19* or Mut2-G4-*H19*. Values are normalized against G3PDH.

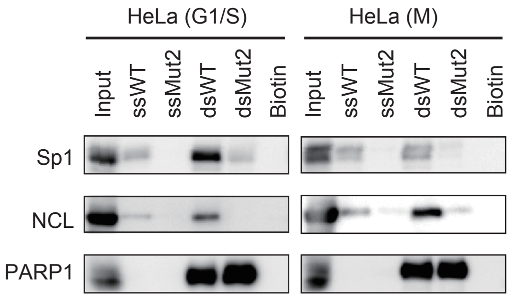

Supplementary Figure 3. The pulldown assays of the *H19* G-quadruplex oligonucleotides using synchronized HeLa cells. HeLa cells were synchronized in G1/S phase by a double-thymidine block. M phase-arrested cells were obtained by the release of double-thymidine block for 8 h, followed by a treatment of the cells with 100ng/ml nocodazole for 3 h. Single-stranded (ss) or double-stranded (ds) biotin-labeled oligonucleotides were incubated with cell lysates and subjected to the pull-down assay. Western blot analysis of Sp1, NCL and PARP1 for the pull-down assay of each oligonucleotide is shown.

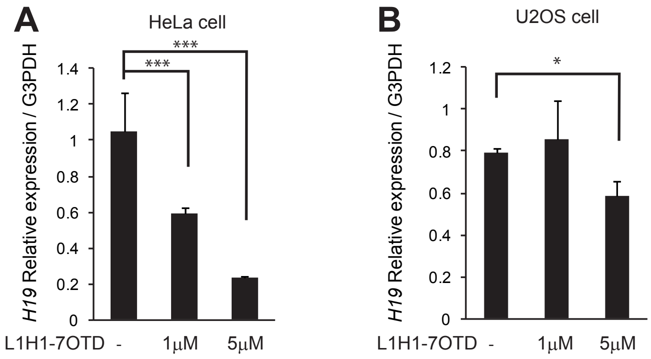

Supplementary Figure 4. L1H1-7OTD suppresses *H19* RNA level in HeLa cells and U2OS cells. HeLa cells (**A**) and U2OS cells (**B**) were incubated with the indicated concentrations of L1H1-7OTD for 2 days. qPCR analyses for endogenous *H19* RNA levels are shown.

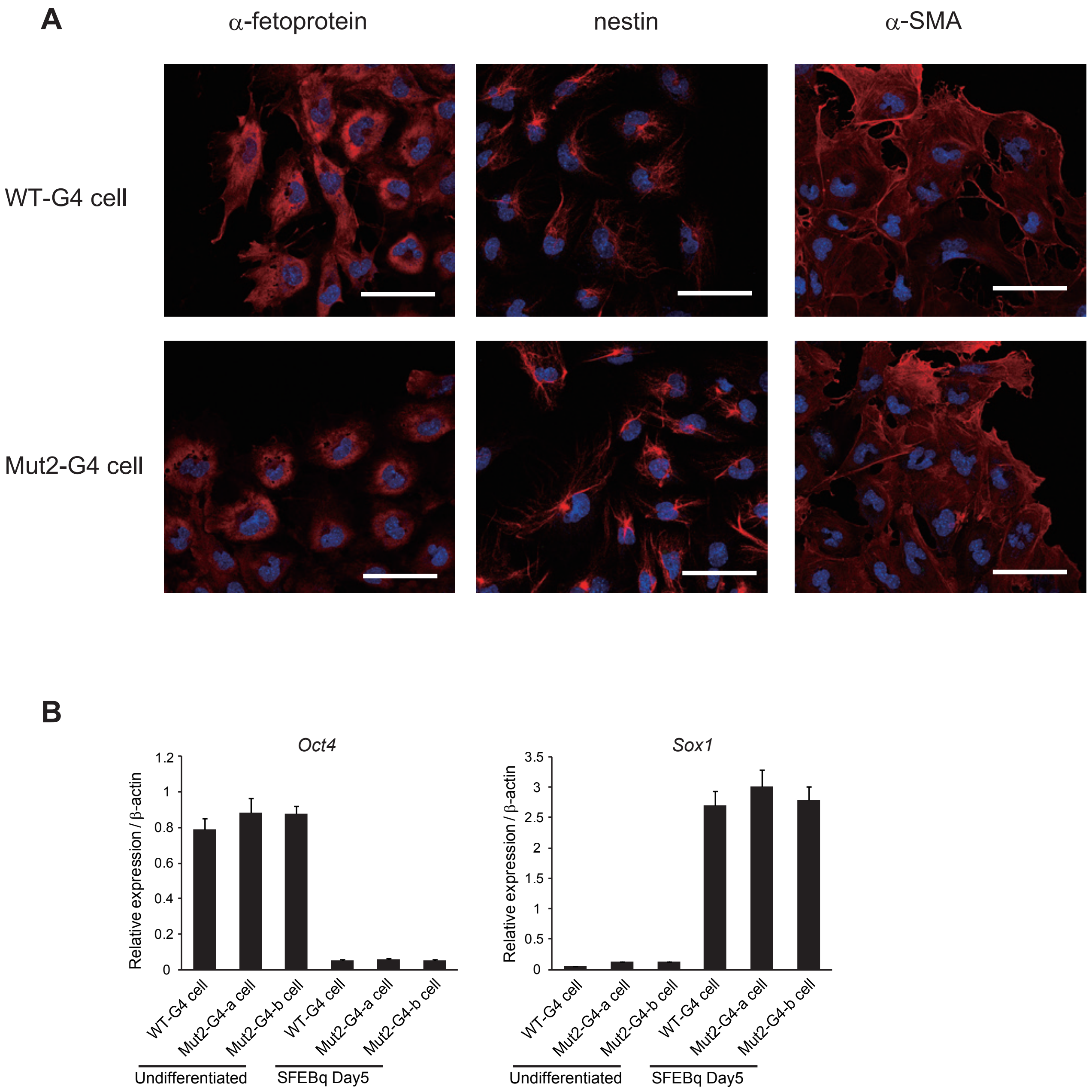

Supplementary Figure 5. Mut2-G4-mESCs retain pluripotency. (**A**) Images of α-fetoprotein, nestin, and α-SMA (red) and Hoechst (bule) in WT-G4 cells and Mut2-G4 cells cultured in EB medium for 6 days. Cells were fixed with 4% (w/v) PFA in PBS at room temperature for 10 minutes, permeabilized with 0.1%(v/v) Triton X-100 in PBS, and stained with primary antibodies in 1% BSA in PBS at 4°C overnight. Antibodies used included anti-α-fetoprotein (R&D Systems, MAB1368), anti-nestin (Santa Crus, sc-58813), and anti-α-SMA (Dako, M0851). Scale bars = 75μm. (**B**) qRT-PCR analysis of RNA levels of *Oct4* and *Sox1* in WT-G4 cells and Mut2-G4 cells in the ESC maintenance medium (undifferentiated) or SFEBq culture on Day 5.

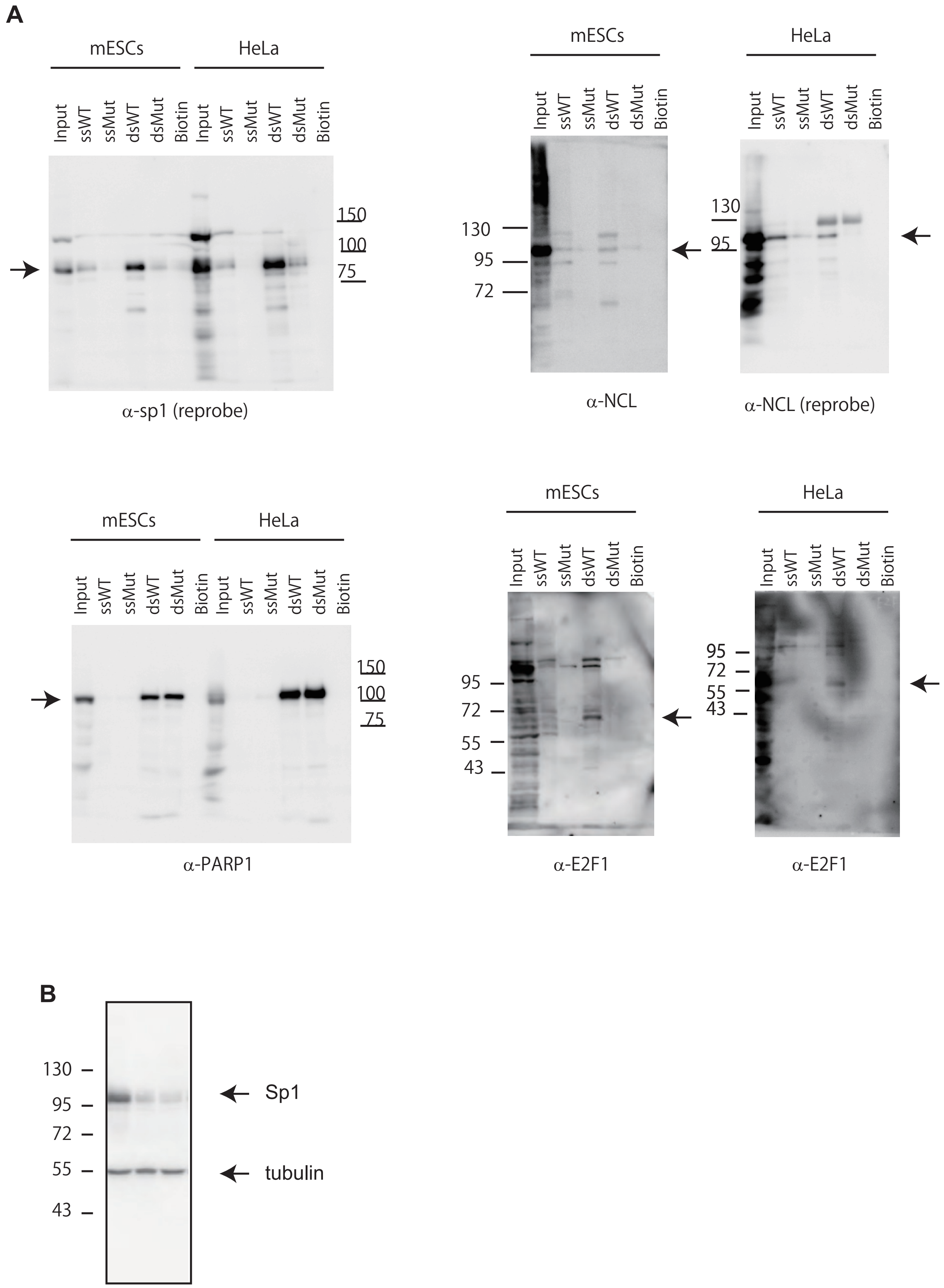

Supplementary Figure 6. Uncropped images of western blot analysis for Figure 5D (A) and Figure 5H (B).
